# Epilepsy and other seizure disorders in acute psychiatric inpatients

**DOI:** 10.1186/s12888-021-03619-y

**Published:** 2021-12-15

**Authors:** Erlend Iversen Nakken, Frithjof Grinde, Arne Vaaler, Ole Kristian Drange, Eylert Brodtkorb, Sverre Georg Sæther

**Affiliations:** 1 Faculty of Medicine and Health Sciences, Norwegian University of Science and Technology, Trondheim, Norway; 2 Department of Acute Psychiatry, Division of Mental Healthcare, St. Olavs University Hospital, Trondheim, Norway; 3 Department of Psychiatry, Sørlandet Hospital HF, Kristiansand, Norway; 4 Department of Neurology and Clinical Neurophysiology, St. Olavs University Hospital, Trondheim, Norway; 5 Department of Neuromedicine and Movement Science, Norwegian University of Science and Technology, Trondheim, Norway; 6 Blue Cross Lade Addiction Treatment Center, Trondheim, Norway

**Keywords:** Epilepsy, Seizures, Acute psychiatric disorders, Acute psychiatry, Prevalence

## Abstract

**Background:**

It is well known that patients with epilepsy have a high rate of psychiatric comorbidity. However, studies exploring epilepsy in psychiatric cohorts are scarce. The aim of this study was to examine the prevalence of seizure disorders in acute psychiatric inpatients.

**Methods:**

This is a cross-sectional study performed in a catchment-area based acute psychiatric department. All patients (age > 18) admitted during September 2011 - March 2012 were eligible for inclusion. Consenting patients were screened for a life-time history of epilepsy or seizures using self-reported questionnaire data and diagnostic codes for epilepsy in hospital and National registries. Patients scoring positive to one or more of these screening criteria underwent a thorough diagnostic validation (chart review), and the seizure disorders were classified as epilepsy, acute symptomatic seizures and/or psychogenic non-epileptic seizures according to current definitions.

**Results:**

A total of 380 out of 591 (64.3%) consecutively admitted patients consented to participate in the study. Eighty-nine patients (23.4%) scored positive to one or more screening criteria. Fifteen (3.9%) were classified with epilepsy, 21 (5.5%) with acute symptomatic seizures and 9 (2.4%) with psychogenic non-epileptic seizures.

**Conclusions:**

This is the first study to report on the prevalence of seizure disorders in acute psychiatric inpatients. The life-time prevalence of epilepsy in this cohort of patients is five – six times as high as reports in the general population. These findings underscore the need for the clinical psychiatrist to have comprehensive knowledge on the interface between epileptology and psychiatry.

**Trials registration:**

ClinicalTrials.gov identifier NCT01415323.

**Supplementary Information:**

The online version contains supplementary material available at 10.1186/s12888-021-03619-y.

## Background

Should psychiatrists care about epilepsy? Epileptic disorders are usually managed by the neurological discipline. However, these disorders are characterized not only by their predisposition to seizures, but also by their neurobiological and psychosocial consequences [1]. The interface between epileptology and psychiatry contains complex and overlapping clinical manifestations [2]. Major imitators of epilepsy such as psychogenic non-epileptic seizures, hyperventilation attacks and convulsive syncope may indeed cause differential diagnostic challenges [3]. Also, the differentiation between seizures in the context of epilepsy and acute symptomatic seizures may be intricate, as seizure precipitants (epilepsy) and provoking factors (acute symptomatic seizures) often are difficult to categorize.

In a recent review, the authors reported that patients with epilepsy have a high prevalence of psychiatric disorders such as current depression (23%), anxiety disorders (20%) and psychotic disorders (5-6%) [4]. These findings have informed the neurologist on the importance of assessing psychiatric comorbidity in patients with epilepsy [5, 6].

However, the opposite is also true. The clinical psychiatrist needs to recognize comorbid seizure disorders in order to inform diagnostic (e.g. organic versus non-organic etiology [7]) and treatment evaluations (e.g. use of medications altering the seizure threshold [8]). Despite its importance, the literature on epilepsy in patients with psychiatric disorders is limited. In fact, the prevalence of epilepsy in patients admitted to acute psychiatric care has never previously been assessed.

The aim of this study was to assess the frequency of epilepsy and other seizure disorders (acute symptomatic seizures and psychogenic non-epileptic seizures) in patients with acute psychiatric disorders. Further, we discuss clinical dilemmas that arise in the diagnosis of seizure disorders from a neurological and psychiatric perspective.

## Methods

### Setting and patients

This is a cross-sectional study performed in the acute psychiatric department at St. Olavs hospital, Trondheim University Hospital, Trondheim, Norway (ClinicalTrials.gov identifier NCT01415323). The department is publicly funded, open to everyone and represents the sole acute psychiatric institution in its catchment area (294,066 inhabitants during the study period). All patients (age > 18) admitted during September 2011 - March 2012 were eligible for inclusion. A total of 380 out of 591 (64.3%) consecutively admitted patients consented to participate in the study.

### Epilepsy screening

All patients were screened for epilepsy or seizures using:

1. Self-reported questionnaire data: “Are you or have you ever been treated for epilepsy?”.
2. Self-reported questionnaire data: “Have you ever had seizures?”.
3. Diagnostic codes for epilepsy (International Classification of Diseases (ICD)-9 (345) or ICD-10 (G40-41)) at the local hospital (available data 1987 – 2020).
4. Diagnostic codes for epilepsy (ICD-9 (345) or ICD-10 (G40-41)) in the Norwegian Patient Registry, which records diagnoses for all patient contacts in the specialist health services in Norway (2007 – 2020).

### Diagnostic validation

We performed a diagnostic validation in all patients scoring positive to one or more of the screening criteria. The diagnoses were classified as epilepsy, acute symptomatic seizures and/or psychogenic non-epileptic seizures by an epileptologist with extensive experience in the field (EB) in consensus with three psychiatrists (AV, OKD and SGS). The diagnostic validation was informed by a review of complete psychiatric and somatic medical records from the local hospital (available data 1987 – 2020), including admission reports, progression notes, discharge reports, electroencephalography (EEG) recordings, brain imaging, and medication history.

A diagnosis of epilepsy was set according to the 2017 revised International League Against Epilepsy criteria [9]. Acute symptomatic seizures and psychogenic non-epileptic seizures were also diagnosed according to current definitions [10–12].

### Definitions

#### Definite epilepsy

Recurrent unprovoked epileptic seizures or at least one unprovoked seizure associated with factors suggesting a high relapse risk [9].

#### Probable (possible) epilepsy

Conditions encompassing one seizure with “risks, but not very high risks, for having another”, as well as for situations with limited information as to the cause of seizures [9].

#### Active epilepsy

Established epilepsy with current treatment or at least one seizure within the last 5 years [13].

#### Epilepsy resolved

Seizure freedom for > 10 years with no treatment for epilepsy > 5 years [9].

#### Acute symptomatic seizure

Epileptic seizures provoked by a clearly identifiable proximate cause, such as evident excessive exposure to drugs and toxic substances [10].

#### Psychogenic non-epileptic seizure

A paroxysmal alteration in motor, sensory, autonomic, or cognitive functions that are not associated with ictal epileptiform activity and occur in the context of psychosocial vulnerabilities [12, 14].

### Group characteristics

Covariables were defined as age, sex, educational status, main diagnosis on admission, substance use and psychotropic medications.

Educational status was categorized into groups consisting of no completion of primary school (< 10 years), high school (13 years), bachelor, master, and Ph. D. degree.

Main diagnosis at admission was categorized into the following ICD-10 diagnostic groups: (1) F00-09 Organic psychiatric disorders, (2) F10-19 Mental and behavioral disorders due to psychoactive substance use, (3) F20-29 Schizophrenia, schizotypal, delusional and other non-mood psychotic disorders, (4) F30-31 Bipolar mood disorders, (5) F32-39 Other mood disorders, (6) F40-99 Other psychiatric disorders, and (7) Other ICD-10 diagnosis outside F00-99.

Substance use was categorized into “benzodiazepines”, “stimulants”, “cannabis”, “alcohol” and “opioids”. This categorization was based on substances consumed during the days/weeks prior to admission. This was evaluated using patient interviews, alcohol breath test and urine analyses of alcohol, benzodiazepines, Z-hypnotics, stimulants, opioids, and cannabis. Patients were categorized as having taken a particular drug prior to admission if they reported use or had a positive urine sample.

Medication on admission was divided into categories of anti-seizure medications, antipsychotics, antihistamines, antidepressants, lithium, stimulants, benzodiazepines, z-hypnotics, and opioids (Additional file 1).

### Statistics

We calculated prevalence estimates with 95% confidence intervals according to Bernoulli trials.

### Ethics

All participating patients gave written informed consent. The study was conducted in accordance with the Helsinki Declaration and approved by The Regional Committee for Medical and Health Research Ethics of Central Norway (2011/137).

## Results

### Prevalence of epilepsy and other seizure disorders

A total of 89 out of 380 (23.4%) patients scored positive to one or more screening criteria for epilepsy or other seizure disorders. Table 1 outlines the percentages of patients scoring positive to the different epilepsy screening criteria.

**Table 1:** Percentages of patients scoring positive to different seizure disorder screening criteria

	Frequencies (% (N) [95% CI])
Positive screen of epilepsy^a^	11.6% (44) [95% CI: 8.4-14.8%]
ICD-9/10 coded data of epilepsy^b^	10.0% (38) [95% CI: 7.0-13.0%]
Self-report of seizures^c^	18.4% (70) [95% CI: 14.5-22.3%]
Self-report of epilepsy^d^	5.0% (19) [95% CI: 2.8-7.2%]

^a^Positive screen of epilepsy encompasses both International Classification of Diseases (ICD)-9/10 coded data and/or self-report of epilepsy

^b^ICD-9/10 coded data for epilepsy represents all encounters for a 345/G40-41 diagnosis in the medical health records from 1987 to 2020

^c^Self-report of seizures was assessed with the following question: “Have you ever had a seizure?”. Missing data in 75 patients (19.7%)

^d^Self report of epilepsy was assessed with the following question: “Are you or have you ever been treated for epilepsy?”. Missing data in 85 patients (22.4%)

**Fig. 1:**
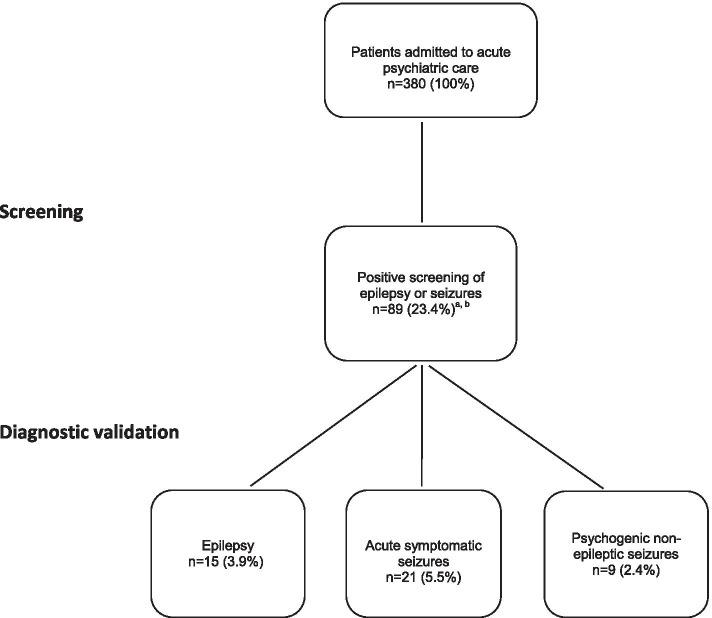
Flow-chart of seizure disorders in 380 patients admitted to acute psychiatric care. ^a^Three patients had combined epilepsy + acute symptomatic seizures; 2 had epilepsy + psychogenic non-epileptic seizures + acute symptomatic seizures (Fig. 2). ^b^Fifty one patients reporting to ever having had epilepsy or seizures had no evidence of seizure disorders in their medical records

**Fig. 2:**
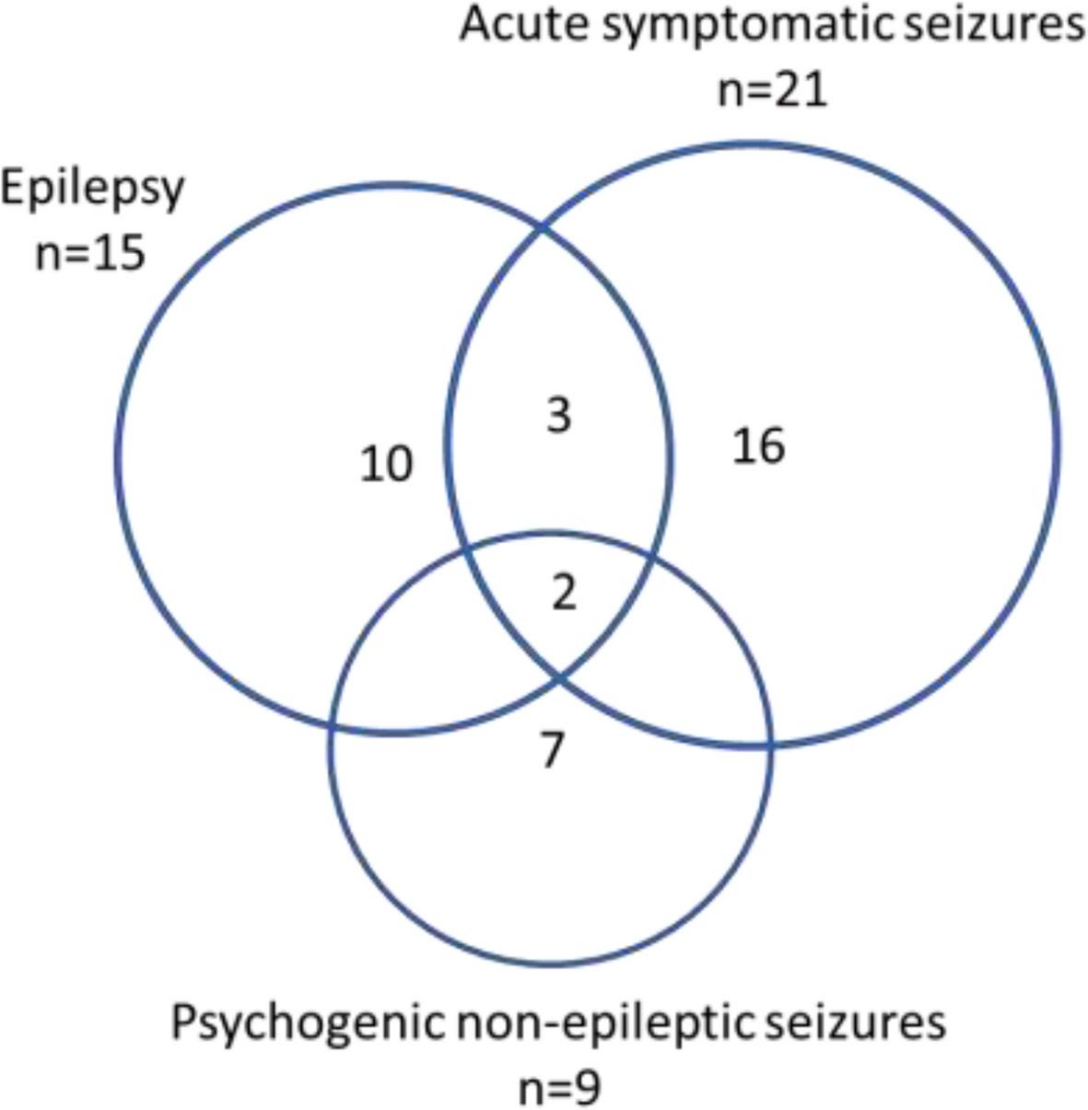
Venn diagram illustrating the distribution and overlap of seizure disorders in acute psychiatric inpatients

After a thorough diagnostic validation of the 89 patients, 15 (3.9%) were diagnosed with epilepsy, 21 (5.5%) with acute symptomatic seizures and 9 (2.4%) with psychogenic non-epileptic seizures (Figs. 1 and 2.). See Table 2 for demographic data of the patients in the diagnostic groups. Three patients with combined epilepsy and acute symptomatic seizures and two patients with combined epilepsy, psychogenic non-epileptic seizures and acute symptomatic seizures in their history are included in these groups. The patients classified without epilepsy or other seizure disorders are described under “Other episodic conditions”.

**Table 2:** Demographic data for patients with different seizure disorders

	Acute symptomatic seizures (*n* = 21)	Psychogenic non-epileptic seizures (*n* = 9)	Definite epilepsy (*n* = 15)	No seizure disorder (*n* = 341)	All (*n* = 380)
Demographics					
Age (Mean (SD))	41 (14)	30 (11)	46 (18)	40 (16)	39 (15)
Female sex (N (%))	6 (28.6%)	8 (88.9%)	4 (26.7%)	167 (49.0%)	184 (48.4%)
Educational status	N (%)	N (%)	N (%)	N (%)	N (%)
Not completed secondary school	0%	0%	0%	0%	0%
Secondary school	13 (61.9%)	4 (44.4%)	4 (26.7%)	147 (43.1%)	166 (43.7%)
High school	8 (38.1%)	4 (44.4%)	8 (53.3%)	145 (42.5%)	161 (42.4%)
Bachelor	0%	1 (11.1%)	3 (20.0%)	40 (11.7%)	44 (11.6%)
Master	0%	0%	0%	7 (2.1%)	7 (1.8%)
Ph. D.	0%	0%	0%	2 (0.6%)	2 (0.5%)
Main diagnosis on admission	N (%)	N (%)	N (%)	N (%)	N (%)
F00-09 Organic psychiatric disorder	0%	0%	2 (13.3%)	16 (4.7%)	18 (4.7%)
F10-19 Disorders related to psychoactive substance use	11 (52.4%)	2 (22.2%)	4 (26.7%)	67 (19.6%)	80 (21.1%)
F20-29 Schizophrenia and other psychotic disorders	1 (4.8%)	1 (11.1%)	0%	49 (14.4%)	51 (13.4%)
F30-31 Bipolar mood disorder	2 (9.5%)	0%	3 (20.0%)	51 (15.0%)	56 (14.7%)
F32-39 Other mood disorder	2 (9.5%)	1 (11.1%)	2 (13.3%)	72 (21.1%)	77 (20.3%)
F40-99 Other psychiatric disorders	4 (19.0%)	5 (55.6%)	2 (13.3%)	74 (21.7%)	84 (22.1%)
Not psychiatric main diagnosis	1 (4.8%)	0%	2 (13.3%)	12 (3.5%)	14 (3.7%)
Substance use^a^	N (%)	N (%)	N (%)	N (%)	N (%)
Benzodiazepines	15 (71.4%)	5 (55.6%)	11 (73.3%)	148 (43.3%)	172 (45.3%)
Stimulants	8 (38.1%)	2 (22.2%)	2 (13.3%)	73 (21.4%)	82 (21.6%)
Cannabis	6 (28.6%)	0%	3 (20.0%)	77 (22.6%)	85 (22.4%)
Alcohol	13 (61.9%)	2 (22.2%)	3 (20.0%)	125 (36.7%)	140 (36.8%)
Opioids	4 (19.0%)	3 (33.3%)	2 (13.3%)	56 (16.4%)	63 (16.6%)
Medications^b^	N (%)	N (%)	N (%)	N (%)	N (%)
Anti-seizure medications	5 (23.8%)	2 (22.2%)	8 (53.3%)	35 (10.3%)	48 (12.6%)
Antipsychotics	7 (33.3%)	2 (22.2%)	7 (46.7%)	105 (30.8%)	118 (31.1%)
Antihistamines	3 (14.3%)	4 (44.4%)	2 (13.3%)	45 (13.2%)	54 (14.2%)
Antidepressants	7 (33.3%)	5 (55.6%)	5 (33.3%)	89 (26.1%)	103 (27.1%)
Lithium	0%	0%	1 (6.7%)	11 (3.2%)	12 (3.2%)
Stimulants	1 (4.8%)	1 (11.1%)	0%	6 (1.8%)	8 (2.1%)
Benzodiazepines	8 (38.1%)	5 (55.6%)	6 (40.0%)	75 (22.0%)	89 (23.4%)
Z-hypnotics	5 (23.8%)	2 (22.2%)	5 (33.3%)	58 (17.0%)	68 (17.9%)
Opioids	2 (9.5%)	1 (11.1%)	0%	16 (4.7%)	19 (5.0%)

^a^Substances included in each category can be found in Additional file 1. Missing data in 11 patients (2.9%)

^b^Information on medications were extracted from medical records from the day of admission. The drugs included in each category can be found in Additional file 1. Missing data in 11 patients (2.9%)

### Epilepsy

The life-time prevalence of epilepsy in this cohort of patients was 15 out of 380 (3.9, 95% CI: [2.0-5.9%]). Out of these 15 patients, 12 were classified as definite epilepsy, whereas three were classified as probable epilepsy according to the International League Against Epilepsy criteria. In the majority, the epilepsy started prior to the inclusion period, whereas two patients got the diagnosis of epilepsy during the observation period of 9 years following index admission.

In the 12 patients with definite epilepsy, six had active epilepsy (four focal, one generalized, one unknown type), and six had epilepsy resolved, the latter of whom five had had self-limited epilepsy of childhood (two absence epilepsy, one epilepsy with centrotemporal spikes, and two undetermined).

The three patients with probable epilepsy were classified as such based on 1) limited clinical information, 2) a seemingly spontaneous seizure with suspected focal epileptiform potentials in the first EEG and 3) a single seizure with recurrent left temporal epileptiform activity persisting during follow-up for 9 years, respectively.

### Acute symptomatic seizures

A history of provoked seizures due to use of substances or medication were reported in the medical records of 21 patients (5.5, 95% CI: [3.2-7.8]), in the majority limited to alcohol or benzodiazepine withdrawal, and in four clearly related to excessive intake of neurotoxic substances. Five out of these patients also had a diagnosis of epilepsy; two had a history of self-limited epilepsy of childhood, two had active focal epilepsy, and one was classified with probable epilepsy.

EEG was recorded in all 16 patients with acute symptomatic seizures alone. All were normal or with mild diffuse unspecific abnormalities, but no epileptiform activity. Brain imaging did not reveal distinct focal changes.

### Psychogenic non-epileptic seizures

A history of events consistent with psychogenic non-epileptic seizures was identified in nine patients (2.4, 95% CI: [0.9-3.9]). Four patients were classified with documented psychogenic non-epileptic seizures (ictal video EEG) according to International League Against Epilepsy-recommended levels of diagnostic certainty [14]. In two patients the diagnoses were clinically established by observation and absence of immediate postictal changes, and in three patients the diagnoses were considered possible/probable by clinical context and characteristic witnessed awake bilateral convulsive movements and no epileptiform interictal EEG findings (hypermotor seizure semiology excluded). Two patients with a history of psychogenic non-epileptic seizures also had acute symptomatic seizures and epilepsy.

### Other episodic conditions

In 16 patients with positive screening for epilepsy, no seizure disorders were identified. Six of these patients were treated with antiseizure medications including clonazepam for psychiatric disorders on admission, and in several a diagnosis of epilepsy had been discussed. Atypical or rapid cycling behavior was identified in eight, syncope in four, panic attacks with hyperventilation in two, and migraine aura in one. EEG findings were unremarkable.

A total of 35 patients reporting a history of seizures in the absence of epilepsy had no evidence of any seizure disorder in their medical records. Several of these patients had clinical notes describing events that could have been misinterpreted (eight had panic attacks with hyperventilation, two had been treated with electroconvulsive therapy, two had syncope, two had Tourette syndrome, one had acute dystonia, and one had withdrawal symptoms with coarse tremor). Nineteen patients had none such alternative causes described in their records.

## Discussion

### Main findings

To the best of our knowledge, this is the first study examining the prevalence of well-validated diagnoses of epilepsy and other seizures disorders in patients admitted to acute psychiatric care. Noteworthy, we found a prevalence five to six times as high (3.9%) as reports from the general population (0.6-0.9% [15, 16]). In addition, we found a history of acute symptomatic seizures and psychogenic non-epileptic seizures in 5.5 and 2.4% of the patients, respectively.

The total prevalence of seizure disorders in our cohort (10%) is comparable to a previous prevalence estimate of seizure disorders in acute psychiatric inpatients [17]. Based on a retrospective chart review Boutros et al. reported that 9.5% of patients admitted to their acute psychiatric center had a diagnosis of epilepsy or “seizure”. However, the authors reported that the charts did not include information that allowed for an accurate diagnosis of epilepsy and noted that the tertiary nature of their facility probably gave inflated estimates.

We screened for epilepsy and seizures using two self-report questions and epilepsy diagnoses in local hospital records and the National Patient Registries. Noteworthy, the most precise screening tool for epilepsy was the self-report question ‘are you or have you ever been treated for epilepsy?” (Table 1). The small overestimation of epilepsy prevalence using this question (5.0% vs. 3.9%) may possibly be related to past or present use of antiseizure medications for psychiatric disorders. The sole use of ICD-9/10 diagnoses from hospital records and registries gave a large overestimation of epilepsy. In fact, only 14 out of 38 patients with a coded diagnosis of epilepsy were found to have true epilepsy on validation. During this validation process it was noted that contacts limited to EEG and clinical appointments for a suspected seizure disorder sometimes had been inappropriately coded as epilepsy. These observations underscore the limitations in solely using registry data as a measure of true epilepsy.

### Differential diagnosis

The epileptic affliction of patients in this clinical sample spanned from a history of drug-resistant and debilitating chronic epilepsies, well-controlled epilepsy, epilepsy resolved (including self-limited childhood epilepsies), as well as recurrent acute symptomatic seizures. The high frequency of past or present epileptic seizures suggests that the brains of many of these people might harbor a shared susceptibility to both psychiatric and seizure disorders possibly by overlapping genetic variants and network abnormalities [18–20].

Patients with psychogenic non-epileptic seizures exhibit a wide range of symptoms; the most common pattern consists of variable movements of limbs, head, and trunk, typically out of phase and often prolonged with preserved consciousness. These conversion symptoms are occasionally associated with self-harm behavior and may be caused by overwhelming and unspoken remote emotional traumas, sometimes related to uncontrolled epilepsy [3, 12]. More than a quarter of patients presenting a seizure disorder was diagnosed with psychogenic non-epileptic seizures; two of which also had a history of epilepsy.

Along with psychogenic non-epileptic seizures, panic attacks with hyperventilation seemed to be the most common condition misinterpreted as an epileptic event. Dizziness and reduced consciousness with loss of control may occur, as well as peripheral sensory and even motor symptoms. The somatic symptoms may be explained by two independent mechanisms taking effect simultaneously, that is, biochemical changes due to lowered pH and cerebral vasoconstriction due to hypocapnia. A provocation test with hyperventilation is usually diagnostic. Hyperventilation may also be a puzzling part of psychogenic non-epileptic seizures [3, 21], whereas conversive elements may be part of the hyperventilation syndrome. The borders between the two conditions may sometimes be blurred.

### Seizure disorders and psychiatric disease

In the 2005 revised International League Against Epilepsy definition of epilepsy, the core elements of the diagnosis were extended beyond recurrent seizures to also include “the neurobiological, cognitive, psychological, and social consequences of the condition”. This definition encompasses a vulnerability to develop psychiatric disorders [1]. The variable temporal relationships between epileptic and psychiatric symptoms in the present series support that these brain-behavior relationships may be bidirectional [2], expressing that psychiatric comorbidity may be part of the epileptic disorder rather than only representing its consequences. Even long resolved self-limited epilepsies of childhood may be associated with poor psychosocial and neuropsychiatric outcome in adult life [22–25], as interestingly was demonstrated in five patients reported here.

This complex patient series illustrates well the obstacles that may take place in the diagnosis, classification and management of seizure disorders in patients with psychiatric comorbidities. Various studies in patients with epilepsy have demonstrated a particular high prevalence of depressive and anxiety disorders substantially contributing to poor health-related quality of life, morbidity, and mortality [26]. It is also well known that psychosis may accompany epilepsy in various ways. Postictal and interictal psychosis usually follow long-standing severe and uncontrolled epilepsy [27]. Although the temporal relationship between psychiatric symptoms and seizure events was not exactly recorded in this study, elements of these complications may have been present in patients with drug-resistant epilepsy. So-called alternative psychoses may occur when seizures suddenly are suppressed by effective treatment. The relationship between epilepsy and interictal psychosis is bidirectional, but epilepsy antedating psychosis is far more common. In a cohort of consecutive patients with both epilepsy (meeting the ILAE diagnostic criteria) and psychosis unrelated to ictal symptoms, 6.8% of patients had developed psychosis prior to the development of epilepsy. Clinical characteristics did not differ in relation to the order of the onset of the two disorders, conceivably due to shared genetic vulnerabilities [28].

Some antiseizure medications may cause various psychiatric adverse events. Moreover, a high load of antipsychotic medications may lower the seizure threshold. All these factors must be considered in patients with comorbid seizures and psychiatric disorders [29]. It has further been hypothesized that various episodic psychiatric symptoms, including panic attacks and violent acts, occasionally may belong to a wider spectrum of epileptic disorders which falls outside the current criteria for epilepsy. Scalp EEGs may be unremarkable or ambiguous, but intracranial EEG may reveal signs of neuronal hyperexcitability, and some of these patients may respond to antiseizure medications [30]. Interestingly, epileptiform and sharp elements in standard EEG recordings have been found to correlate with a higher frequency of hypomanic episodes in bipolar II disorder even in the absence of a history of seizures [31].

Patients with substance use disorders are at high risk of developing seizures, both during the withdrawal phase (e.g. alcohol) and during intoxications (e.g. stimulants). These acute symptomatic seizures are provoked by systemic insults outside physiological limits and contrasts to the seizures precipitated by everyday circumstances in patients with epilepsy. Seizure occurrence may be dependent on the individual seizure threshold, either inherently [20, 32] or facilitated by otherwise subclinical subtle brain lesions [33] or influenced by prescribed psychotropic treatment, sleep deprivation and stress, particularly when these factors are combined. It has been reported that substance use disorders are considerably more common in patients with epilepsy as compared to the general population [34]. However, the borders between epilepsy and acute symptomatic seizures may be blurred [35], and epilepsy can easily be over-diagnosed. To complicate the picture even more, epilepsy and acute symptomatic seizures may occur in the same individual, as in five patients in this patient series. In these patients, acute symptomatic seizures differed from previous habitual seizures by the presence of evident withdrawal symptoms or intoxications with neurotoxic substances.

Noteworthy, inflammatory brain diseases should always be ruled out in patients presenting with acute psychiatric symptoms and epileptic seizures. Indeed, we advocate prompt diagnostic procedures for autoimmune or infectious encephalitis in such patients to ensure early appropriate treatment [36, 37].

### Strengths and limitations

A strength of the present study is that the study sites (the acute psychiatric department and neurological department) are the sole institutions providing acute psychiatric care and epilepsy management in the catchment area. The rather small sample size allowed for a detailed review of the entire medical record of each patient with a strict validation procedure using established definitions for epilepsy and other seizure disorders. Nevertheless, despite these efforts, the precision of exact diagnoses of seizure disorders might represent a problem in this type of retrospective study, as the differentiation between recurrent unprovoked seizures (epilepsy) and repeated acute symptomatic seizures may be challenging. Moreover, a large part of the self-reported seizure events was not addressed in the medical records. This might have led us to underestimate the number of patients with acute symptomatic seizures [33]. The inclusion rate of 64.3% compares favorably to other studies in acute psychiatric settings [38–40]. However, since patients with more severe acute psychiatric symptoms are more prone to decline study participation, this numbers may represent a selection bias.

## Conclusions

A history of seizure disorders is frequent in patients admitted to acute psychiatric care and may substantially add to the burden of stigma and disability. Seizures may manifest as epilepsy, acute symptomatic seizures or psychogenic non-epileptic seizures and should be carefully investigated, as these disorders need different therapeutic approaches. This is the first study to report on the prevalence of these disorders in patients admitted to acute psychiatric care.

The findings underscore the need for a comprehensive interdisciplinary management of these patients. We suggest more joint research and educational efforts between the fields of neurology and psychiatry to broaden the understanding of the multifaceted challenges occurring in patients with combined psychiatric and seizure disorders.

## Supplementary Information

**Additional file 1: Table S1** Drugs included in each medication category. **Table S2** Drugs included in each substance use category.

## Data Availability

The datasets used and/or analysed during the current study are available from the corresponding author on reasonable request.

## Abbreviations

EEG: Electroencephalography
ICD: International Classification of Diseases
