# Supplementary material for: Epilepsy and other seizure disorders in acute psychiatric inpatients
Source: BMC Psychiatry. 2021 Dec 15;21:626. doi: 10.1186/s12888-021-03619-y (PMC8672464; doi:10.1186/s12888-021-03619-y)
Supplement: Supplementary file 1 — Additional file 1: Table S1 Drugs included in each medication category. Table S2 Drugs included in each substance use category. [file 12888_2021_3619_MOESM1_ESM.docx]

**Additional files 1**

***Table S1:*** *Drugs included in each medication category.*

| Medications category | Drugs included |
| --- | --- |
| Anti-seizure medication | Levetiracetam, lamotrigine, gabapentine, pregabaline, valproate, carbamazepine, zonisamide |
| Antipsychotics | Quetiapine, aripiprazole, clozapine, olanzapine, risperidone, paliperidone, ziprasidone, zuclopenthixol, flupentixol, levomepromazine, perphenazine, chlorprothixene, |
| Antihistamines | Alimemazine, hydroxyzine, cetirizine, promethazine, desloratadine, loratadine, ranitidine |
| Antidepressants | Citalopram, escitalopram, sertraline, duloxetine, paroxetine, fluoxetine, venlafaxine, buproprione, mirtazapine, klomipramine, amitriptyline, trimipramine, doxepin, mianserin, phenelzine |
| Lithium | Lithium |
| Stimulants | Methylphenidate, dexamphetamine, lisdexamphetamine |
| Benzodiazepines | Ooxazepam, diazepam, clonazepam, alprazolam, alopam, nitrazepam, flunipam, clobazam, lorazepam |
| Z-hypnotics | Zopiclone, zolpidem |
| Opioids | Morphine, oxycodone, codeine, tramadol, buprenorphine, methadone |

| Substance use category | Drugs included |
| --- | --- |
| Benzodiazepines | Oxazepam, desmethyldiazepam, nitrazepam, flunitrazepam, clonazepam, alprazolam |
| Stimulants | Amphetamine, methamphetamine, cocaine, 3,4-methylendioxymethamphetamine, 3,4-methyldioxyamphetamine |
| Cannabis | Cannabis |
| Alcohol | Ethanol |
| Opioids | Morphine, codeine, ethylmorphine, methadone, buphrenorphine, oxycodone |

***Table S2:*** *Drugs included in each substance use category.*
